# Genetic and Environmental Influences on Chinese Language and Reading Abilities

**DOI:** 10.1371/journal.pone.0016640

**Published:** 2011-02-10

**Authors:** Bonnie Wing-Yin Chow, Connie Suk-Han Ho, Simpson Wai-Lap Wong, Mary M. Y. Waye, Dorothy V. M. Bishop

**Affiliations:** 1 University of Oxford, Oxford, United Kingdom; 2 City University of Hong Kong, Hong Kong, Special Administrative Region, People's Republic of China; 3 The University of Hong Kong, Hong Kong, Special Administrative Region, People's Republic of China; 4 The Hong Kong Institution of Education, Hong Kong, Special Administrative Region, People's Republic of China; 5 School of Biomedical Sciences, The Chinese University of Hong Kong, Hong Kong, Special Administrative Region, People's Republic of China; King's College London, United Kingdom

## Abstract

This study investigated the etiology of individual differences in Chinese language and reading skills in 312 typically developing Chinese twin pairs aged from 3 to 11 years (228 pairs of monozygotic twins and 84 pairs of dizygotic twins; 166 male pairs and 146 female pairs). Children were individually given tasks of Chinese word reading, receptive vocabulary, phonological memory, tone awareness, syllable and rhyme awareness, rapid automatized naming, morphological awareness and orthographic skills, and Raven's Coloured Progressive Matrices. All analyses controlled for the effects of age. There were moderate to substantial genetic influences on word reading, tone awareness, phonological memory, morphological awareness and rapid automatized naming (estimates ranged from .42 to .73), while shared environment exerted moderate to strong effects on receptive vocabulary, syllable and rhyme awareness and orthographic skills (estimates ranged from .35 to .63). Results were largely unchanged when scores were adjusted for nonverbal reasoning as well as age. Findings of this study are mostly similar to those found for English, a language with very different characteristics, and suggest the universality of genetic and environmental influences across languages.

## Introduction

Children show great individual variation in language and reading skills [1]. Both hereditary and environmental factors play a role in the origins of these individual differences, but their relative contributions can differ for various language and reading skills as demonstrated in twin studies [2]. To date, twin studies have been conducted on alphabetic languages, mainly on English. Thus, whether these roles of heredity and environment are universal or differ across languages remains unknown. Chinese has very different linguistic characteristics compared with alphabetic scripts. So, the investigation of the genetic influences on Chinese learning will lead to enhanced understanding of, not only the nature of Chinese acquisition, but also the universal or specific factors of language and reading acquisition across languages. We investigated the genetic and environmental origins of Chinese reading and related language skills in 312 Chinese twin pairs in Hong Kong.

### Cognitive skills important to reading abilities

Reading development of children is interwoven with language development and is supported by various cognitive skills including awareness in phonology, morphology and orthography [3]. Awareness in this context is generally conceptualized as the ability to manipulate language or reading units. Phoneme, morpheme and grapheme are the smallest sound, meaning and print units of a language or a script. Thus, phonological, morphological, and orthographical awareness refer to the ability to manipulate sound, meaning and print units respectively. In English, the bulk of studies have focused on strong positive links between reading abilities and phonological awareness as assessed by tasks such as removing the onset (initial sound) from a word, e.g. ‘break’ becomes ‘rake’, or removing the rime (final sound), e.g. ‘break’ becomes ‘bray’ [4], [5]. Rapid naming ability, assessed by speeded naming of symbols such as color patches, numbers, letters or pictures, is another skill with strong links to English reading, which also involves phonological processing [6]. Phonological memory, i.e. immediate repetition of strings of sounds, is also correlated with English reading [7], but often does not contribute uniquely over phonological awareness and rapid naming in predicting reading ability [8]. However, as noted by Tunmer and Nesdale, phonological skills are necessary but not sufficient for reading acquisition [9]. Converging research evidence has shown that morphological awareness [10] and orthographic skills [11] make a unique contribution to English reading after phonological skills have been taken into account.

In contrast to the many studies on the cognitive features of English acquisition, relatively little is known about Chinese language and reading abilities. Because Chinese has very different characteristics compared with English, many of the research findings about English may not apply in Chinese. Chinese is a morphosyllabic language in which each character, the primary unit of writing, represents both a syllable and a lexical morpheme. A character and its components (strokes or radicals) do not represent phonemes. For instance, the character 洋 (ocean) and its phonetic radical 羊 represent the syllable /joeng4/. These one-to-one-to-one relations among Chinese characters, syllables and morphemes promote a high degree of metalinguistic prominence pertaining to syllables [12] and morphemes [13]. Also, there is extensive lexical compounding, e.g., the word 電視 [*/din6 si6/*; *television*] is composed of the morphemes 電 [*/din6/*; *electric*] and 視 [*/si6/*; *vision*]. The large number of homophonic morphemes further underscores the importance of morphological awareness in learning Chinese [14]. Furthermore, Chinese is a tone language; thus a Chinese syllable in different tones (six tones in Cantonese) represents different meanings and each of them is denoted by a different character. Although there is no symbol in a character that signifies tone, this awareness of tone is essential for distinguishing the meaning of a particular syllable, which allows the correct identification of a character [15].

It is sometimes assumed that written Chinese, unlike English, has arbitrary relationships between orthography and phonology, but this is not the case. In Chinese, the phonological radical of a character provides a cue to pronunciation, but it is ambiguous. Only around 40% of the Chinese characters can be successfully decoded by the orthography-phonology correspondence rules, because this is relevant only to semantic-phonetic compound characters which contain phonetic radicals that provide useful information in reading the characters [16]. So, learning of Chinese relies heavily on forming relatively arbitrary associations between printed and spoken language. There are around 4500 regularly used characters [17], so a large number of phonological units have to be stored in and retrieved from the lexicon during the process of learning Chinese. This explains why the tasks of rapid automatized naming, which reflects the speed of access to the lexicon, and phonological memory are linked to Chinese reading ability [18], [19].

Regarding Chinese orthography, each Chinese character occupies a fixed square space in print, and its components and radicals have habitual positions. Knowledge of correct orientation of orthographic units aids the formation of character representations in the lexicon, and locating cues of pronunciation and meaning of characters. These orthographic skills are prominent in reading Chinese [20]. In general, past research has indicated that morphological awareness [21], phonological awareness at the syllable and onset-rime levels [12], [22], tone awareness [15], rapid automatized naming [18], phonological memory [19], and orthographic skills [20] contribute to Chinese language and reading skills. The causes of individual variations of these cognitive skills in Chinese were examined in this study.

### Twin study

Individual variations in language and reading skills are influenced both by heredity (e.g., genes linked to language and reading skills, such as KIAA0319) [23] and environmental factors (e.g., effects of schooling) [24]. Investigations of how heredity and environment influence language and literacy skills have used the twin design, which tests sources of twins' resemblance based on the difference of monozygotic (MZ) and dizygotic (DZ) twins' genetic relatedness. In general, MZ twins share all their genes, while DZ twins on average share the same alleles for half of their segregating genes.

Phenotypic variance reflects the influence of genetic factors plus that of environmental factors (including measurement errors), and any interaction between these. Provided that we assume environmental similarity is equivalent for MZ and DZ twin pairs, any extra similarities of MZ twins over DZ twins can be attributed to genetic origins. The contribution of genetic factors can be quantified by heritability (*h* ^2^). Environmental factors include shared environment (*c* ^2^) which makes twins resemble each other, such as parenting practice and family socioeconomic status, and nonshared environment (*e* ^2^) which distinguishes twins from one another, such as illnesses that only one twin experienced and twin-specific peer groups. Strong shared environmental influences are indicated when the similarity between co-twins of MZ twin pairs and that of DZ twin pairs is high and equivalent. The twin design has been employed to study the acquisition of speaking and reading alphabetic languages, mainly English. Robust research evidence has indicated strong genetic and negligible to small environmental influences on reading skills [25], [26], and rapid naming ability [27], [28]. Moreover, converging research evidence has shown that genetic factors, compared to environmental factors, exert relatively greater influences on phonological memory [29], [30], phonological awareness [2], [31], and orthographic skills [32]. However, shared environmental influences are relatively greater for vocabulary knowledge [33], [34], and grammar/morphological skills [35], [36].

The growing twin research in alphabetic languages has contributed to better understanding of the roles of heredity and environment in the development of these languages, mainly English. However, to the best of our knowledge, there is a lack of twin study on languages which have very different linguistic characteristics compared with alphabetic ones, such as Chinese. This study investigates how hereditary and environmental factors determine the development of Chinese language and reading skills, and their related cognitive skills, in 312 pairs of Chinese twins.

## Methods

### Ethics statement

Ethical approval was obtained from the Human Research Ethics Committee for Non-clinical Faculties of the University of Hong Kong. Parental written consent was obtained for each participant.

### Participants

We recruited 339 pairs of typically developing Chinese twins who were a) aged from 3 to 11, b) of the same sex in each pair (i.e., male-male pair or female-female pair), and c) using Cantonese as their mother tongue. They were recruited through kindergartens and primary schools in Hong Kong, and parental consent was obtained for each participant. In general, children in Hong Kong enter kindergarten and primary school at around age 3 and age 6 respectively, and are provided with 3 years of kindergarten education and 6 years of primary education. Most Hong Kong children start learning to read and write Chinese characters at a very young age and the majority start learning to read and write words at age 4 or below: 98.4% and 91.9% for learning to read and write respectively [37]. Zygosity of the twin pairs was determined by SNP testing. All children were given an audiometric screening test to ensure they had normal-range hearing for speech frequencies. Five twin pairs with unreliable results in audiometric screening test were excluded. We also excluded children who could not hear 35dB or above with the better ear, thereby yielding a sample of 312 twin pairs. There were 228 pairs of monozygotic twins (116 male pairs and 112 female pairs) and 84 pairs of same-sex dizygotic twins (50 male pairs and 34 female pairs) in our final sample.

The high proportion of MZ to DZ twins is consistent with the fact that the DZ to MZ twinning ratio tends to be lower in Asian than in Western populations [38]. The same-sex dizygotic to monozygotic twin ratio in our sample was 0.37. With opposite-sex twin pairs included, the dizygotic to monozygotic twin ratio was around 0.65 for twins born to Chinese fathers or mothers in Singapore from 1986 to 2001 [39]. Assuming there is an equal number of same-sex and opposite-sex dizygotic twin pairs, the same-sex dizygotic to monozygotic twin ratio was around 0.33. So, the proportion of twin types in our study was comparable to that of the population prevalence. Table 1 shows the age, sex and zygosity distribution of the participants.

**Table 1:** Age distribution of the participants.

	Frequency (Twin pairs)	Percent (%)
**Age**		
3–4	17	5.45
4–5	57	18.27
5–6	58	18.59
6–7	43	13.78
7–8	47	15.06
8–9	49	15.71
9–10	21	6.73
10–11	20	6.41
Total	312	100.00
**Sex**		
Boy	166	53.21
Girl	146	46.79
Total	312	100.00
**Zygosity**		
MZ	228	73.08
DZ	84	26.92
Total	312	100.00

### Measures

Our sample covered a wide age range, 3 to 11 years, but all measures were constructed to be suitable for children across this age range, and all analyses were conducted on age-adjusted scores.

#### Word reading

A 48-item character reading list and 150 items adapted from the reading subtest of the Hong Kong Test of Specific Learning Difficulties in Reading and Writing (HKT-SpLD) [40] were combined. The HKT-SpLD is a standardized test developed for Hong Kong primary school children, and items in the reading subtest are common two-character words of Grade 1 to Grade 6 levels. The words are arranged in an order of increasing difficulty. Children were required to read each word aloud. Testing stopped when they failed to read 15 consecutive items. Kindergartners started from the character reading list, and were given the items adapted from the HKT-SpLD if they progressed beyond this list. However, the first item of the HKT-SpLD reading subtest was regarded as the entry point for all primary school children. They were given the kindergarten character reading list only if they failed to read the first 15 consecutive items on the HKT-SpLD reading subtest. The maximum score was 198, and Cronbach's alpha was .996.

#### Receptive vocabulary

The receptive vocabulary test consisted of 2 practice trials and 80 test trials translated and adapted for Chinese from the Peabody Picture Vocabulary Test – Fourth Edition (PPVT-IV) [41]. For each trial, the experimenter read out the target item and the child was required to select a picture from the four options to match it. An entry point for each grade level and a basal rule were set according to pilot testing on 90 kindergartners and junior primary school students. The basal rule was fulfilled if correct responses were given in nine or all trials in the first 10 consecutive trials from the corresponding entry point. Testing stopped when the child failed 11 or all trials in 12 consecutive trials. The maximum score was 80, and Cronbach's alpha was .96.

#### Phonological memory

A nonword repetition task consisted of a series of nonword strings ranging from two syllables to seven syllables. A nonword string was constituted by Cantonese syllables and had no lexical meaning as a whole (e.g., 點土/*dim2 tou2*/). There were two practice trials and 14 test trials. For each trial, the child was presented a nonword string, in which the inter-syllable interval was 0.5 second, by an mp3 player. The child was then requested to repeat the nonword string in the exact order of syllables presented, and the response was recorded. For a nonword string, a point was given for each correct syllable, and also for each correct pair of consecutive syllables, but a point was deducted for each excessive syllable. Testing stopped when the child failed four consecutive items. The maximum score was 124, and Cronbach's α was .90.

#### Tone awareness

The Cantonese tone task consisted of 3 practice trials and 15 test trials, administered with a computer. There were three blocks of five test trials arranged in the following order: three-syllable, two-syllable, and one-syllable blocks, because the more syllables given could provide more cues on identifying the correct tones and were thus relatively easier. For each one-syllable trial, three pictures were shown, each illustrating a syllable. Each syllable had a different tone. The child was required to name each picture, and was told the name if they were not able to name it correctly. This procedure was to ensure that the child knew the syllables represented by the pictures before proceeding to the actual tone test. Then, a meaningless sound with a given tone was presented, and the child was asked to select the picture representing the syllable which had the same tone. For instance, a meaningless Cantonese first tone sound (i.e., high-level tone sound) was presented, and three pictures illustrated a pig (豬 /*zyu1*/), a lock (鎖 /*so2*/) and a letter (信 /*seon3*/) respectively were shown. The answer was a pig (豬 /*zyu1*/) which has a Cantonese first tone. The two- and three- syllable trials had the same procedure, except each picture illustrated a two- or three- syllable word and the sound of two or three tones was presented. The maximum score was 15, and Cronbach's α was .66.

#### Syllable and rhyme awareness

The syllable deletion task consisted of three blocks of trials in an increasing difficulty order: real words, nonwords and nonsense words. A real word was formed by combining Cantonese syllables and had lexical meaning (e.g., 望遠鏡 /*mong6 jyun5 geng3*/ [binoculars]). A nonword was composed of Cantonese syllables and had no lexical meaning as a whole (e.g., 女任綠 /*neoi5 jam6 luk6*/). A nonsense word was created from nonsense syllables that conformed to the phonological constraints of Cantonese, and neither the nonsense syllables nor the compound as a whole had lexical meaning (e.g., /*fou2 moi1 peng5*/). The items were orally presented by the experimenter and the children were required to produce an answer orally with one syllable taken away from the compound words. In each block, two trials required deletion of the first syllable, two trials required deletion of the last syllable, and one trial required deletion of the middle syllable. For example, the real word ‘望遠鏡’ /*mong6 jyun5 geng3*/ (binoculars) without ‘遠’/*jyun5*/ is ‘望鏡’ /*mong6 geng3*/. The target answers of some real word items were meaningful words. The maximum score was 15.

The rime detection task consisted of two practice trials and nine test trials. For each item, the experimenter read out a target syllable, and then read out three syllables and simultaneously showed three pictures illustrating each of them. The child was required to select a syllable from the three options which rhymed with the target syllable. The options and the target syllable rhymed with the same tone in some items, but they had different tones in other items. For example: ‘人’ /*jan4*/ (human) was read out as the target syllable, and ‘牙’ /*ngaa4*/ (tooth), ‘猴’ /*hau4*/ (monkey) and ‘雲’ /*wan4*/ (cloud) were then presented with their illustrations. The answer was ‘雲’ /*wan4*/ (cloud) which rhymed with the target syllable ‘人’ /*jan4*/ (human). The maximum score was nine. The maximum score of the combined task was 24, and Cronbach's α was .88.

#### Rapid automatized naming

The speeded number naming task consisted of six rows of five digits [2], [ 4], [ 5], [ 7], [ and 9]. These digits were arranged in different orders for each row. The child had to name all digits at the fastest speed possible. Two trials were completed, and the averaged time was recorded. The measure for analyses was (1/average time), so a higher score indicated a better rapid automatized naming ability, proportional to number of words read per second.

#### Morphological awareness

Three tasks of morphological awareness arranged in an order of increasing difficulty were administered. Testing stopped when the child failed four out of five consecutive items. The receptive morphological awareness task consisted of two practice trials and 10 test trials. For each item, five pictures were presented to the children simultaneously and the experimenter read out the target item which represented a novel concept created by combination of morphemes. The child was required to select a picture from the five options which illustrated the target item. For example, in one trial of this task, the target item was a striped elephant (斑象 /*baan1 zoeng6*/) which represented a new concept of an elephant with stripes on its body. Five pictures showing a) a zebra (斑馬/*baan1 maa5*/), b) a striped dog (斑狗 /*baan1 gau2*/), c) stripes and an elephant (斑+象/*baan1*/+/*zoeng6*/), d) a dog and an elephant (狗+象 /*gau2*/+/*zoeng6*/), and e) a striped elephant (斑象 /*baan1 zoeng6*/), were presented. The maximum score of this task was 10.

The morphological construction task consisted of one practice trial and 12 test trials. For each item, a scenario was orally presented by the experimenter, and the child was asked to actively construct words for the newly presented objects or concepts according to the scenarios. The practice trial was aided with illustration. One test trial is indicated below as an example. “An island that is full of yellow chrysanthemums, is called a yellow chrysanthemum island (黃菊島 /*wong4 guk1 dou2*/). What will we call the island which is full of red peach blossom?” The target answer was a red peach blossom island (紅桃島 /*hung4 tou4 dou2*/). A target answer was awarded two points, and partially correct answers (e.g., a peach blossom island, 桃島 /*tou4 dou2*/) were each awarded one point. The maximum score of this task was 24.

The homophone task consisted of one practice trial and five test trials. For each trial, a character was orally presented in the context of a word, and the child was required to produce as many words including this character as possible in 10 seconds. The child was then asked to produce as many words including the homophones of this character as they could in 10 seconds. For example, in one trial of the task, the target character was ‘兒’ of the word ‘兒童’ (children /*ji4 tung4*/). The words constituting this character could be ‘兒子’ (son /*ji4 zi2*/), ‘兒歌’ (children’s song /*ji4 go1*/) etc., while the words composed of its homophones could be ‘姨媽’ (aunt /*ji4 maa1*/), ‘懷疑’ (suspect /*waa14 ji4*/) etc. Two points were given for at least one correct word produced in both parts, while one point was awarded for at least one correct word given in one part only. The maximum score of this task was 10. The score of the combined task was 44, and Cronbach's α was .90.

#### Orthographic skills

**Figure 1:**
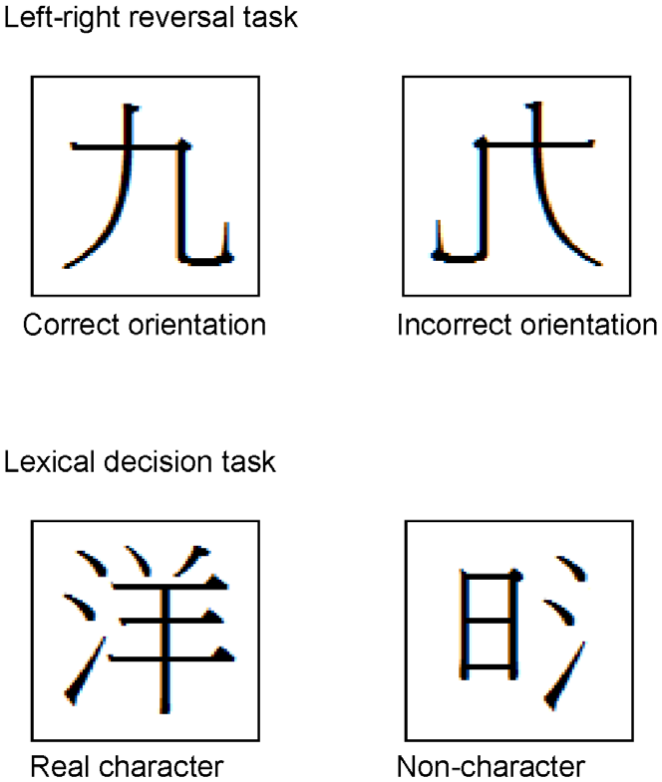
Constructed items illustrating the left-right reversal and the lexical decision tasks.

Two tests of orthographic skills adapted from the Hong Kong Test of Specific Learning Difficulties in Reading and Writing (HKT-SpLD) [49] were used. The left-right reversal task assessed the knowledge of correct orientation of highly frequent orthographic units in Chinese characters. This task consisted of 21 simple Chinese characters and 4 Arabic numbers, of which 14 were left-right reversed. The child was presented all items simultaneously arranged in a five by five matrix, and required to cross out items with an incorrect orientation with a pencil. The lexical decision task assessed the knowledge of Chinese character structure. This task consisted of 30 rare real Chinese characters, and 30 noncharacters with radicals placed in illegal positions. All of them were left-right structured and composed of two radicals. A real left-right structured Chinese character normally had a semantic radical on the left and a phonetic radical on the right. A noncharacter was the combination of two semantic radicals, two phonetic radicals, or a semantic radical and a phonetic radical in their illegal positions, all of which were illegal in Chinese character structure. The child was presented items arranged in 12 rows of five items each on two separate pages, and was required to cross out the noncharacters. A point was given for an item identified correctly,i.e., an incorrect oriented item/noncharacter was crossed out or a correct oriented item/real character was left uncrossed. Example items illustrating these two tasks constructed by the authors are shown in Figure 1. The maximum score of the combined task was 85, and Cronbach's α was .93.

#### Nonverbal reasoning

The 36-item Raven's Coloured Progressive Matrices (RCPM) [42] was employed to assess nonverbal reasoning. As this task has not yet been normed on the Chinese population, raw scores were used. The two trial items were not included in the total score. The maximum score was 34, and Cronbach's α was .93. To adjust for age effects, the raw scores of RCPM were regressed on children's age, and the standardized residuals represented children's nonverbal reasoning ability.

### Procedure

Each child was tested in a 1-hour session by trained psychology major undergraduates and graduates in their school, their home, or our research laboratory in Hong Kong according to parents' preference. Saliva was collected from co-twins with DNA kits for zygosity assessment.

## Results

Standardized scores adjusted for age were employed in the analyses. These scores indicated how many standard deviations a participant's performance was above or below the mean of all participants of that age (i.e., a standardized score of 1 represents 1 standard deviation above the mean). Phenotypic bivariate correlations were computed on the scores of a cotwin randomly selected in each twin pair. The phenotypic bivariate correlations of word reading with the other tasks were all significant (*p*s<.05), indicating significant links between word reading and these tasks.

To reduce the effects of extreme scores on model fitting, standardized scores that exceeded absolute score of 3 (i.e., performance exceeded 3 standard deviation above or below the mean) were censored, by replacing them with 3 or −3 as appropriate. Results of independent sample t-tests on the scores of a cotwin randomly selected in each twin pair showed MZ twins and DZ twins did not differ significantly on any tasks (*p*s>.05). However, to minimize the possible effects of different performance between MZ and DZ twins on model fitting, all scores were standardized by zygosity. We did not adjust scores for sex, because sex differences on the measures were small and well below the size specified by McGue and Bouchard as large enough to impact estimates of heritability [43].

Adjusted scores were first fitted to the full ACE model, and then nested AE or CE models, of each variable by OpenMx using the R statistical modeling package [44]. We subsequently tested model fit after additionally adjusting scores for nonverbal ability.

### ACE models

Results indicated that all models provided a satisfactory goodness of fit (*p*s>.05). Table 2 shows the intraclass correlations by zygosity and the estimates of genetic and environmental influences on age-adjusted scores. Strong and significant genetic influences were found on word reading, phonological memory and tone awareness, with nearly three-fourths of the variance in word reading and around half of the variances in phonological memory and tone awareness explained by genetic influences. Genetic factors also exerted moderate and significant influences on rapid automatized naming and morphological awareness. In contrast, strong influences of shared environment were indicated in receptive vocabulary and syllable and rhyme awareness, with around three-fifths of their variances explained by shared environmental influences. Shared environmental factors also exerted moderate influences on orthographic skills and modest but significant effects on phonological memory. Effects of nonshared environment, which included measurement errors, were moderate across tasks, except word reading, where they were negligible.

**Table 2:** Intraclass correlations by zygosity, genetic model parameter estimates, and nested model statistics of each variable controlling for age.

	Intraclass Correlations		ACE Models			Nested Models		
Variable	MZ	DZ	a^2^	c^2^	e^2^	Best model	*Δχ^2^*(Δ*df* = 1)	*p*
Word reading	.90	.54	.73(.54,.92)	.18(−.02,.38)	.09(.08,.11)	AE	1.31	.25
Receptive vocabulary	.68	.62	.11(−.06,.29)	.57(.38,.75)	.32(.28,.36)	CE	0.87	.35
Phonological memory	.75	.51	.50(.28,.71)	.25(.03,.46)	.26(.22,.29)	ACE	–	–
Tone awareness	.55	.29	.52(.23,.82)	.02(−.26,.29)	.46(.40,.52)	AE	0.01	.93
Syllable and rhyme awareness	.71	.67	.08(−.08,.23)	.63(.47,.80)	.29(.25,.33)	CE	0.48	.49
Rapid automatized naming	.58	.37	.42(.15,.70)	.15(−.11,.41)	.43(.37,.48)	AE	0.60	.44
Morphological awareness	.64	.42	.44(.19, .69)	.20(−.04,.44)	.36(.31,.41)	AE	1.20	.27
Orthographic skills	.55	.45	.20(−.05,.45)	.35(.11,.58)	.45(.40,.51)	CE	1.36	.24

*Note*. Number of twin pairs ranged from 210 to 228 for MZ and from 80 to 84 for DZ. All ACE models had a satisfactory goodness of fit indicated by a nonsignificant *χ^2^*change between the saturated and the ACE models (*Δχ^2^* ranged from 0.73 to 7.25, *Δdf* = 6, *p*s>.05). MZ = Monozygotic twins; DZ = Dizygotic twins; a^2^ = additive genetic variance; c^2^ = shared environment variance; e^2^ = nonshared environment variance; *Δχ^2^* and *Δ*df are the differences between the ACE and the nested models.

(95% confidence intervals in parentheses).

To indicate the extent of genetic and environmental contributions to these skills when general cognitive ability have been taken into account, scores controlled for age and nonverbal reasoning were fitted to the models. Table 3 shows the intraclass correlations by zygosity and the estimates of genetic and environmental influences on scores controlling for age and nonverbal reasoning. In general, similar patterns of relative genetic and shared environmental contributions were obtained, except the shared environmental influences on phonological memory and the genetic influences on morphological awareness became nonsignificant when nonverbal reasoning was controlled for.

**Table 3:** Intraclass correlations by zygosity, genetic model parameter estimates, and nested model statistics of each variable controlling for age and nonverbal reasoning.

	Intraclass Correlations		ACE Models			Nested Models		
Variable	MZ	DZ	a^2^	c^2^	e^2^	Best model	*Δχ^2^*(Δ*df* = 1)	*p*
Word reading	.85	.52	.68(.48, .89)	.17(−.04, .38)	.15(.13, .17)	AE	1.15	.28
Receptive vocabulary	.54	.50	.09(−.15, .32)	.45(.23, .68)	.46(.40, .52)	CE	0.27	.60
Phonological memory	.71	.46	.51(.28, .75)	.20(−.03, .43)	.29(.25, .33)	AE	1.25	.27
Tone awareness	.51	.22	.49(.40, .59)	.00(.00, .00)	.51(.44, .57)	AE	0.00	1.00
Syllable and rhyme awareness	.57	.53	.07(−.15, .30)	.50(.28, .71)	.43(.37, .49)	CE	0.23	.63
Rapid automatized naming	.58	.37	.42(.15, .70)	.15(−.11, .41)	.43(.37, .49)	AE	0.58	.45
Morphological awareness	.48	.36	.25(−.03, .53)	.23(−.03, .49)	.52(.45, .59)	AE	1.30	.25
						CE	1.72	.19
Orthographic skills	.45	.43	.06(−.21, .32)	.40(.15, .64)	.55(.47, .62)	CE	0.09	.76

*Note*. Number of twin pairs ranged from 210 to 227 for MZ and from 80 to 84 for DZ. All ACE models had a satisfactory goodness of fit indicated by a nonsignificant *χ^2^*change between the saturated and the ACE models (*Δχ^2^* ranged from 0.81 to 6.77, *Δdf* = 6, *p*s>.05). MZ = Monozygotic twins; DZ = Dizygotic twins; a^2^ = additive genetic variance; c^2^ = shared environment variance; e^2^ = nonshared environment variance; *Δχ^2^* and *Δ*df are the differences between the ACE and the nested models.

(95% confidence intervals in parentheses).

### Nested models

The non-significant parameter (either A or C) of the ACE models was dropped and the model fit of the nested models (AC or AE models) was compared to that of the full ACE models to further clarify the roles of genetics and environment in the aforementioned skills. The model fitting statistics are shown in Tables 2 and 3. For age-adjusted scores, nested models with the C parameter dropped resulted in a non-significant decrease in model fit for word reading, tone awareness, rapid automatized naming and morphological awareness. Also, nested models with the A parameter removed did not reduce model fit significantly for receptive vocabulary, syllable and rhyme awareness and orthographic skills. In general, the nested models showed a stronger effect of either A or C (e.g., a stronger effect of A than C for word reading). However, for some measures, the confident intervals of a^2^ and c^2^ overlapped (e.g., orthographic skills), and so these nested models should be interpreted cautiously.

These nested model analyses were re-run on age- and nonverbal reasoning- adjusted scores. In addition, a nested model with the C parameter dropped was tested for phonological memory and that with the A parameter dropped was tested for morphological awareness. The removal of these paths all yielded a nonsignificant decrease in model fit. For morphological awareness, the AE and the CE models did not have poorer model fit than the full ACE model, but the model fit decreased substantially when the A parameter or the C parameter was dropped in the AE and the CE models (i.e., the E model). Therefore, when the effects of age and nonverbal reasoning were accounted for, it was not possible to distinguish between genetic or shared environmental influences as causes of twin-twin similarity in morphological awareness.

## Discussion

This study allowed us to quantify the relative roles of genetic and environmental influences in various Chinese language and reading skills, giving results that were partly consistent with findings on alphabetic languages.

### The role of nonverbal ability

Before discussing how our results compare with those of studies on English-speaking children, it is important to consider the role of nonverbal ability. As noted by Brooks et al, most cognitive tests show a ‘positive manifold’ of inter-correlations [45]. Plomin and Kovas more recently have stressed the extent to which different cognitive skills have common genetic influences from ‘generalist genes’ [46]. This suggests that when we find, for instance, a strong genetic influence on reading ability, we may be simply picking up a general genetic influence on intelligence, rather than a more specific influence on literacy. In general, our findings indicated that this was not the case. When the effects of nonverbal reasoning were taken into account, results were similar to those obtained without IQ control, except the shared environmental influences became nonsigificant for phonological memory, and both genetic and shared environmental influences could contribute comparably to morphological awareness.

### Comparison with previous data on English-speaking samples

The stronger influences of genetic factors on Chinese word reading, phonological memory and rapid automatized naming, and the greater effects of shared environmental factors on Chinese receptive vocabulary, agree with past research evidence on English children [25], [29], [32]. They suggest these genetic or environmental contributions are universal across languages, even for those with very different characteristics. Table 4 shows a comparison of estimates in past twin studies and the present Chinese twin study.

**Table 4:** A comparison of heritability estimates of language and reading skills in past twin studies and the present Chinese twin study.

	Past twin study			Chinese
Skills	Authors	Country	Heritability estimate	Heritability estimate
Word reading	Byrne et al., 2005^a^	Australia, USA	.70	.73/.68^CA^
	Byrne et al., 2007^a^	Australia, USA	.49 to .72	
	Byrne et al., 2009^a^	Australia, USA, Scandinavia	.82	
	Samuelsson et al., 2007^a^	Australia, USA, Scandinavia	.61 to .91	
	Samuelsson et al., 2008^a^	Australia, USA, Scandinavia	.33 to .84	
	Brooks et al., 1990^b^	USA	.45^CA^	
	Gayàn & Olson, 2003^b^	USA	.85^CA^	
	Keenan et al., 2006^b^	USA	.66.65^CA^	
	Hart, Petrill, Thompson, & Plomin, 2009^c^	USA	.45 to .94	
	Petrill et al., 2006^c^	USA	.68.67^CA^	
	Harlaar, Dale, & Plomin, 2005^d^	UK	.63 to .74	
Vocabulary	Byrne et al., 2002^a^	Australia, USA, Norway	.18	.11/.09^CA^
	Byrne et al., 2009^a^	Australia, USA, Scandinavia	.44	
	Samuelsson et al., 2005^a^	Australia, USA, Scandinavia	.32	
	Hart, Petrill, DeThorne, et al., 2009^c^	USA	.29 to .49	
	Mather & Black, 1984^e^	USA	.68	
	Van Hulle et al., 2004	USA	.08 to .20	
	Dionne et al., 2003^d^	UK	.10 to .21	
Phonological memory	Byrne et al., 2002^a^	Australia, USA, Norway	.19	.50/.51^CA^
	Samuelsson et al., 2005^a^	Australia, USA, Scandinavia	.57	
	Wadsworth et al., 1995^b^	USA	.95	
	Kovas, Hayiou-Thomas, et al., 2005^d^	UK	.41	
Phonological awareness	Byrne et al., 2002^a^	Australia, USA, Norway	.52	.08/.07^CA^
	Byrne et al., 2005^a^	Australia USA	.63	
	Samuelsson et al., 2005^a^	Australia, USA, Scandinavia	.60	
	Gayàn & Olson, 2003^b^	USA	.83^CA^	
	Petrill et al., 2006^c^	USA	.48.30^CA^	
	Petrill et al., 2007^c^	USA	.14 to .59	
	Hohnen & Stevenson, 1999	UK	.52 to .62	
	Kovas, Hayiou-Thomas, et al., 2005^d^	UK	.38	
Rapid Automatized Naming	Byrne et al., 2002^a^	Australia, USA, Norway	.00 to .66	.42/.42^CA^
	Byrne et al., 2005^a^	Australia, USA	.60	
	Samuelsson et al., 2005^a^	Australia, USA, Scandinavia	.64	
	Davis et al., 2001	USA	.62	
	Hart, Petrill, Thompson, & Plomin, 2009^c^	USA	.42 to .79	
	Petrill et al., 2006^c^	USA	.77.72^CA^	
Morphology/Grammar	Byrne et al., 2002^a^	Australia, USA, Norway	.31	.44/.25^CA^
	Samuelsson et al., 2005^a^	Australia, USA, Scandinavia	.29	
Orthographic skills	Gayàn & Olson, 2003^b^	USA	.87^CA^	.20/.06^CA^

*Note*. Studies with typically-developing samples in the age range of 3 to 11 years are included in this table. Studies based on the same sample are denoted by the same superscript in the Authors column. In case of studies examined the same variable based on the same sample, the study with the largest sample in the analyses is included. Superscript CA denotes estimates with general cognitive ability accounted for. References for this table are given in Text S1.

Our findings also suggest some language-specific influences. The results indicated genetic factors exerted a strong influence on Chinese tone awareness. This is a novel and language-specific finding because tones represent lexical meanings in Chinese, and it is a unique characteristic which English does not possess. Our estimates of heritability of tone awareness resemble those found in English for phonological awareness.

On the other hand, Chinese phonological awareness at syllable and rhyme levels was more affected by shared environment than by genes. Past studies have highlighted the prominence of genetic influences on English phonological awareness in kindergarteners [2], junior elementary school children [31], and older children [32]. These converging links did not extend to Chinese phonological awareness. Even though our phonological awareness task did not involve any written language, we suggest that the ambiguous print-sound correspondence of Chinese may exert an effect. In English, there is evidence that people use knowledge of what the written word looks like when doing phonological awareness tasks [47]; our study suggests that when such orthographic knowledge is not a reliable cue to phonological structure, children need specific training, and this shows up as an environmental effect in the analysis. Thus for children learning Chinese, phonological awareness development may have heavier reliance on how far this skill is specifically taught. Phonological scripts can be employed in learning Chinese, but there is geographical diversity in use of the different methods: Pinyin in mainland China, Zhu Yin Fu Hao in Taiwan, and lack of systematic teaching on Cantonese phonetics in Hong Kong. These may explain why shared environmental effects on phonological awareness are stronger in Chinese than those in English.

Regarding orthographic skills, these too showed negligible genetic influence in Chinese, in contrast to strong genetic influence in English. The smallest orthographic units in Chinese are unpronounceable strokes which combine to form stroke pattern, such as radicals. Orthographic units in Chinese are visually more complex, and more numerous than letters in English. Also, stroke patterns are put together in a square frame, unlike letters which always arrange from left to right in English letter strings. There are positional regularities for radicals in a character that form the rules of legal character structure. To acquire the knowledge of orientations and positions of stroke patterns and rules of forming legal characters, Chinese children rely on various methods, such as copying and writing [22], which may contribute to relative stronger environmental influences on the development of orthographic skills.

### Limitations and future studies

Findings of this study should be interpreted in the context of two caveats. First, the present study included children of a relatively wide age range, which covered children in different stages of language and reading development. In future work we will report findings from a follow-up study of the same sample, which will provide information on the extent to which genetic and environmental influences change with age. Also, the degree of genetic and environmental overlap between language and reading skills is a promising area of future research. Second, our sample size is relatively small, particularly for DZ twins, and so confidence intervals around estimates were large, and so only large effects would be detected. Nevertheless, it is encouraging that our findings are in general consistent with those from English-speaking samples, except where there are good reasons to suppose that language-specific factors may come into play.

### Implications and conclusion

To conclude, this study has extended past twin research on language and reading abilities in alphabetic languages to Chinese. There are several important implications of our findings. First, the present study has indicated the relative roles of genetic and environmental influences in Chinese language and reading abilities. These findings are useful in facilitating future investigations of the root causes of Chinese language and reading difficulties, and providing insights on structuring effective Chinese language and literacy learning environments for children. Second, by comparing our findings with those of past studies on English abilities, the universal or specific factors of language and reading acquisition across languages could be understood. Specifically, the genetic contributions to word reading, phonological memory and rapid automatized naming, and the shared environmental influences on receptive vocabulary, are likely to be universal across languages. However, the importance of shared environment on phonological awareness at the syllable and rhyme levels is unique to Chinese. Lastly, we indicated that genetic factors played a prominent role in certain Chinese skills, including word reading, tone awareness, phonological memory and rapid automatized naming. These are helpful for future molecular genetics studies, especially in selecting target language or reading skills for investigation in Chinese populations.

## Supporting Information

Text S1 **References for** **Table 4** **.**
(DOC)
