# Supplementary material for: Genetic and Environmental Influences on Chinese Language and Reading Abilities
Source: PLoS One. 2011 Feb 10;6(2):e16640. doi: 10.1371/journal.pone.0016640 (PMC3037369; doi:10.1371/journal.pone.0016640)
Supplement: Text S1 — References for Table 4 . (DOC) [file pone.0016640.s001.doc]

Text S1. References for Table 4.

Brooks A, Fulker DW, DeFries J (1990). Reading performance and general cognitive ability: A multivariate genetic analysis of twin data. PERS INDIV DIFFER 11(2): 141-146.

Byrne B, Coventry WL, Olson RK, Samuelsson S, Corley R, et al (2009) Genetic and environmental influences on aspects of literacy and language in early childhood: Continuity and change from preschool to grade 2. J NEUROLINGUIST 22(3): 219-236.

Byrne B, Delaland C, Fielding-Barnsley R, Quain P, Samuelsson S, et al (2002) Longitudinal twin study of early reading development in three countries: Preliminary results. ANN DYSLEXIA 52: 49-73.

Byrne B, Samuelsson S, Wadsworth S, Hulslander J, Corley R, et al. (2007) Longitudinal twin study of early literacy development: Preschool through grade 1. READ WRIT 20(1-2): 77-102.

Byrne B, Wadsworth S, Corley R, Samuelsson S, Quain P, et al (2005) Longitudinal twin study of early literacy development: Preschool and kindergarten phases. SCI STU READ 9(3): 219-235.

Davis CJ, Knopik VS, Olson RK, Wadsworth SJ, DeFries JC (2001) Genetics and environmental influences on rapid naming and reading ability. ANN DYSLEXIA 51: 231-247.

Dionne G, Dale PS, Boivin M, Plomin R (2003) Genetic evidence for bidirectional effects of early lexical and grammatical development. CHILD DEV 74(2): 394-412.

Gayán J, Olson RK (2003) Genetic and environmental influences on individual differences in printed word recognition. J EXP CHILD PSYCHOL 84(2): 97-123.

Harlaar N, Dale PS, Plomin R (2005) Telephone testing and teacher assessment of reading skills in 7-year-olds: II. Strong genetic overlap. READ WRIT 18(5): 401-423.

Hart SA, Petrill SA, DeThorne LS, Deater-Deckard K, Thompson LA, et al. (2009) Environmental influences on the longitudinal covariance of expressive vocabulary: Measuring the home literacy environment in a genetically sensitive design. J CHILD PSYCHOL PSYC 50(8): 911-919.

Hart SA, Petrill SA, Thompson LA, Plomin R (2009). The ABCs of math: A genetic analysis of mathematics and its links with reading ability and general cognitive ability. J EDUC PSYCHOL 101(2): 388-402.

Hohnen B, Stevenson J (1999) The structure of genetic influences on general cognitive, language, phonological, and reading abilities. DEV PSYCHOL 35(2): 590-603.

Keenan JM, Betjemann RS, Wadsworth SJ, DeFries JC, Olson RK (2006) Genetic and environmental influences on reading and listening comprehension. J RES READ 29(1): 75-91.

Kovas Y, Hayiou-Thomas ME, Olive, B, Dale PS, Bishop DV, et al. (2005) Genetic influences in different aspects of language development: The etiology of language skills in 4.5-year-old twins. CHILD DEV 76(3): 632-651.

Mather PL, Black KN (1984) Hereditary and environmental influences on preschool twins. DEV PSYCHOL 20(2): 303–308.

Petrill SA, Deater-Deckard K, Thompson LA, DeThorne LS, Schatschneider C (2006) Reading skills in early readers: Genetic and shared environmental influences. J LEARN DISABIL-US 39(1): 48-55.

Petrill SA, Deater-Deckard K, Thompson LA, Schatschneider C, Dethorne LS, et al. (2007) Longitudinal genetic analysis of early reading: The Western Reserve Reading Project. READ WRIT 20(1-2): 127-146.

Samuelsson S, Byrne B, Olson RK, Hulslander J, Wadsworth S, et al. (2008) Response to early literacy instruction in the United States, Australia, and Scandinavia: A behavioral-genetic analysis. LEARN INDIVID DIFF 18(3): 289-295.

Samuelsson S, Byrne B, Quain P, Wadsworth S, Corley R, et al (2005) Environmental and genetic influences on prereading skills in Australia, Scandinavia, and the United States. J EDUC PSYCHOL 97(4): 705-722.

Samuelsson S, Olson R, Wadsworth S, Corley R, Defries JC, et al (2007) Genetic and environmental influences on prereading skills and early reading and spelling development in the United States, Australia, and Scandinavia. READ WRIT 20(1-2): 51-75.

Van Hulle CA, Goldsmith H, Lemery KS (2004) Genetic, environmental, and gender effects on individual differences in toddler expressive language. J SPEECH LANG HEAR R 47(4): 904-912.

Wadsworth, SJ, DeFries, JC, Fulker, DW, Olson, RK, et al (1995) Reading performance and verbal short-term memory: A twin study of reciprocal causation. INTELLIGENCE 20(2): 145-167.
